# Self-Healing Properties of Water Tree with Microcapsule/Cross-Linked Polyethylene Composite Material Based on Three-Layer Core-Shell Structure

**DOI:** 10.3390/polym16111445

**Published:** 2024-05-21

**Authors:** Bo Zhu, Xinyu Tao, Hao Sun, Yaqi Zhu, Shengkun He, Ximu Han

**Affiliations:** MOE Key Laboratory of Engineering Dielectrics and Its Application, Harbin University of Science and Technology, Harbin 150080, China; 2320310226@stu.hrbust.edu.cn (X.T.); 2220310174@stu.hrbust.edu.cn (H.S.); zyqher@163.com (Y.Z.); 2220310197@stu.hrbust.edu.cn (S.H.); 2220310194@stu.hrbust.edu.cn (X.H.)

**Keywords:** cross-linked polyethylene, microcapsule, water tree, self-healing, dielectric properties, nano-SiO_2_

## Abstract

To overcome the degradation of insulating properties caused by the water tree aging of cross-linked polyethylene (XLPE), a self-repairing material for XLPE based on a microcapsule system is proposed. Three-layer shell nucleus microcapsules/XLPE composites with different microcapsule doping content are prepared. The water tree aging experiments are carried out using the water-needle electrode method to analyze the ability of microcapsules to repair the damaged areas of water trees. The results show that, compared with the XLPE material without microcapsules, the electrical properties of composites decline significantly when the doping concentration of three-layer shell nucleus microcapsules is large. When the doping concentration is 1.0 wt%, the microcapsule/XLPE composite breakdown strength has no noticeable change, and the dielectric loss factor does not change significantly, the space charge density decreases, and the space charge properties have been improved considerably. When the water tree branch develops to the position where the microcapsules are located, the microcapsules will rupture and release their internal repair materials and catalysts and react with water to produce an organic silicone resin to fill the water tree cavity, which can achieve an excellent self-healing effect. In addition, the nano-SiO_2_ on the surface microcapsules can make the microcapsules and matrix better integrated, which avoids the microcapsule accumulation that tends to occur when incorporating microcapsules, thus improving the repair rate.

## 1. Introduction

With the acceleration of China’s modernization process, the power grid extensively uses XLPE insulation due to its good performance. Before XLPE is widely used in cables, PE was used. PE has excellent low-temperature resistance, resistance to most acids and alkalis, and excellent electrical insulation properties. PE has non-polar characteristics, so it has a low loss and conductive strength of the characteristics of the large, so it is generally used as a high-voltage power cable insulation production material. XLPE is PE with high-energy rays or cross-linking agents under the action of its macromolecules for producing cross-linking production. XLPE has almost the same the electrical properties as PE. Compared to PE, it is greatly improved in terms of physical properties as well as chemical properties. At the same time, heat resistance is significantly improved. Therefore, XLPE power cables have the advantages that PE power cables cannot be compared with: lightweight, high heat resistance, strong load capacity, and good insulation performance [1,2]. However, in the process of manufacturing and laying, the cable will inevitably cause certain microscopic defects; the operation will be subjected to local high temperatures, the role of industrial frequency voltage, and different substances in the soil of various degrees of corrosion [3,4]. Therefore, microscopic defects will also occur in the XLPE cable. Under the continuous action of water in the surrounding environment, the tiny defects in the insulation layer are very prone to water tree aging [5,6]. Water trees in solid insulation are dendritic hairline insulation defects formed by micron-sized holes and nanometer-sized channels and are a permanent material degradation phenomenon that occurs within the insulation. Under high electric fields, moisture gradually accumulates in the XLPE material, forming water tree channels. Water tree channels cause a decrease in the dielectric strength of the material, resulting in partial discharges and material damage; the water tree is the leading cause of damage to high-voltage power cables [7,8]. The water branches keep growing along the direction of the electric field force and begin to bifurcate to form water tree regions, some of which gradually diffuse into electric branches under the action of a high-frequency electric field or overvoltage, causing electric field distortion and the eventual breakdown of the cable insulation layer [9,10]. At present, the method of injecting repair fluid into the water tree aging cable is usually used to repair it. Still, this method requires that the repaired part has moisture and that the cable needs power outage treatment, and the effect in practical application is not ideal. Meanwhile, the repair solution also needs to be under the action of the catalyst to react with water, and the inconsistency of the diffusion rate of the two will also lead to a decrease in the repair effect [11,12,13].

Currently, self-healing systems are divided into two main categories: intrinsic and extrinsic [14]. Intrinsic self-healing mainly utilizes its own characteristics for self-healing, relying on reversible valence bond changes and chemical reactions to change the intrinsic chemical structure of the material to achieve the repair of damage. According to the different reaction mechanisms, they can be categorized into reversible covalent bond reaction type and reversible non-covalent bond reaction type [15]. Intrinsic self-healing systems are difficult to apply to insulating materials because valence bonding reactions alter the chemical structure of the substrate and severely deteriorate its intrinsic properties. The extrinsic self-healing material is a composite material formed by a foreign material and a matrix for the purpose of self-healing the matrix. The main practice nowadays is to bury the sealed repair agent into the raw material, and in case of damage, the defects will damage the encapsulated structure, which will release the repair agent and complete the self-healing process. Extrinsic self-healing systems mainly include microcapsules, bionic blood vessels, and hollow fibers [16]. Microcapsule self-healing systems not only have good stability and high repair rates but also have little impact on the performance of the matrix [17]. Therefore, a microcapsule self-healing system was selected to realize the self-healing of insulating materials. The use of microcapsules for the repair of insulating materials is promising, but further challenges remain: (1) Repair agents are usually triggered by a single stimulus, such as heat, pH, or catalysts, leading to premature failure of microcapsules in complex environments. (2) The microcapsule self-healing system inevitably introduces defects into the insulating material, resulting in some degradation of the insulating properties of the composite material. (3) The distribution of microcapsules in the matrix is random, and it is difficult to carry out directional and gradient distribution.

Microcapsule self-healing technology was first proposed by White et al. It refers to wrapping the repair material in a specific microcapsule and incorporating the microcapsules into the matrix material. When a damage occurs in the matrix, the stress tip of the damaged part will cause the microcapsules to rupture. The internal repair agent flows out and rapidly solidifies under the action of external stimulus conditions, filling the aging area and realizing the self-healing process of the material [18,19]. He Jinliang et al. designed a microcapsule/epoxy composite material for repairing electric trees, which utilized the free radicals generated during the electrical aging process as initiators to achieve self-repair of electric trees without external condition stimulation [20,21]. Li et al. conducted a study on the repair of electric tree damage by doping urea formaldehyde resin (UF)/dicyclopentadiene (DCPD) microcapsules within low-density polyethylene and determined that the composite material doped with 1 wt% microcapsules had better insulation strength and better self-healing effect [17,22]. Sima Wenxia and other scholars have studied magnetic field and ultraviolet dual response microcapsules. By adding magnetic nanoparticles to the repair material as a magnetic target, a microcapsule that can be oriented and moved under the action of an external magnetic field is synthesized so that the epoxy composite material has a repeatable and highly oriented self-healing ability [23,24]. Zhang Yanfang et al. developed a kind of PUF-SiO_2_/DCPD microcapsule modified by a nano-SiO_2_ surface. Compared with the traditional PUF/DCPD microcapsules, the mechanical properties and thermal stability of the modified microcapsules were improved. In addition, the dispersion of PUF-SiO_2_/DCPD microcapsules in polyethylene matrix is better, and the effect of aging damage repair is further enhanced [25]. When the insulating material undergoes water tree aging, the microcapsule will rupture under the action of the electric field, and the repair material inside it will react with the water in the water tree branches under the action of the catalyst to produce organic matter [26,27]. However, most of the current self-repair methods based on microcapsule systems use single-layer shell nucleus microcapsules, which will limit the water tree repair effect due to the different positions of the repair solution and the catalyst [28,29]. Moreover, when the microcapsules are mixed with XLPE, agglomeration easily occurs, and the microcapsules cannot be ruptured and cannot repair aged areas in time, resulting in a decrease in repair rate. Therefore, it is necessary to develop a three-layer shell nucleus microcapsule with nano-SiO_2_ surface modification [30], which has both the repair solution and catalyst inside to ensure the accuracy and timeliness of water tree self-healing.

This paper focuses on exploring the self-healing method of XLPE material water tree aging. Therefore, the urea–formaldehyde resin modified by nano-SiO_2_ (UF@SiO_2_) was used as the wall material, and the repair solution dodecyltrimethoxysilane (DTMS) and catalyst linear alkylbenzene sulfonic acid (LABSA) were selected as the core material. A microcapsule with a three-layer shell nucleus structure was prepared by interfacial polymerization, and the preparation method of the three-layer microcapsules/XLPE composite material was explored. The electrical properties of the prepared three-layer shell nucleus microcapsules/XLPE composites were tested and analyzed for the change mechanism, and the optimum percentage content of the microcapsules in XLPE composites was obtained. In addition, the water tree aging test of the composites proved the inhibition ability of microcapsules on water tree growth and the self-healing ability after aging.

## 2. Experiment

### 2.1. Preparation of the Three-Layer Shell Nucleus Microcapsules

In this paper, a two-step method was adopted to prepare microcapsules, in which UF prepolymer was designed as the wall material of microcapsules. Then, the wall material was used to wrap the core material to form a single-layer shell nucleus microcapsule, after which UF prepolymer modified by nano-SiO_2_ (20 nm) and the three-layer shell nucleus microcapsules were prepared by a similar method. The preparation process has good reliability and stability, and the formed microcapsules have uniform morphology. The preparation process is below.

(1) Preparation of UF prepolymer modified by nano-SiO_2_: After the urea was fully solved with deionized water, 37 percent concentration of the formaldehyde solution and nano-SiO_2_ was added and thoroughly mixed. Triethanolamine (TEA) was slowly added to the mixed solution to adjust the pH until 8~9, and then it was stirred at 70 °C for one hour. After the response, it was cooled to room temperature. The obtained white solution with a certain viscosity is a UF prepolymer modified by nano-SiO_2_ (UF@SiO_2_).
(2) Fabrication of single-layer shell nucleus microcapsules: The emulsifier SDBS, catalyst LABSA, and deionized water were fully combined and stirred for half an hour at 40 °C under the water bath. When the system showed a white emulsion, sodium hydroxide (NaOH) solution was slowly added dropwise to increase the pH of the system to 9~10. After the pH was stable, UF water resistance modifier resorcinol, UF curing agent ammonium chloride, and UF prepolymer were added dropwise to the solution. Then, the citric acid was used to reduce the pH of the solution to about 3.0 slowly, and it was kept stirring at 53 °C for three hours. After the reaction, the sample was washed and dried to obtain the single-layer shell nucleus microcapsules.
(3) Preparation of three-layer shell nucleus microcapsules: The repair solution DTMS, emulsifier SDBS, and single-layer shell nucleus microcapsules were thoroughly mixed in deionized water and stirred for half an hour at 40 °C under the water bath. Then, UF water resistance modifier resorcinol, UF curing agent ammonium chloride, and UF@SiO_2_ prepolymer were added sequentially. After stirring for 3 min, the pH of the mixed solution was gradually reduced to 3.0 with a dilute hydrochloric acid solution. Additionally, the process of adjusting pH should be controlled within 10~20 min. After the pH of the solution was stabilized, it was heated to 53 °C. Under this condition, the reaction was kept for 3 h. After the reaction, the sample was washed and dried to obtain the three-layer shell nucleus microcapsules.

**Figure 1:**
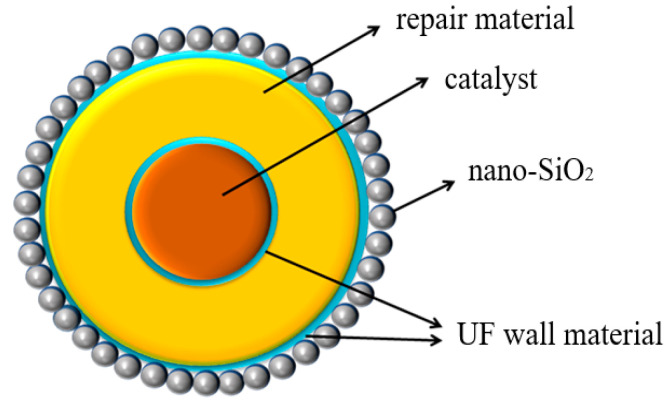
Structure model of three-layer shell nucleus microcapsules.

The internal structure of three-layer shell nucleus microcapsules is shown in Figure 1, which shows that nano-SiO_2_ is attached to the surface of the capsule wall. The internal core materials are single-layer shell nucleus microcapsules and the repair solution DTMS. In addition, the catalyst LABSA exists in the single-layer shell nucleus microcapsule. Since the repair solution needs to be catalyzed by the catalyst to hydrolyze and condense with water, the quality proportion of DTMS to LABSA inside microcapsules should be controlled at 4:1 to ensure that the reaction is entirely carried out. To improve the stability of microcapsules in matrix processing and operation, the amount of the ammonium chloride and the resorcinol should account for 1% and 2% of the prepolymer, respectively.

### 2.2. Fabrication of Three-Layer Shell Nucleus Microcapsules/XLPE Composite Material

The LDPE was put into a mixing machine (product of Harper Electric Technology LLC, Harbin, China, model RM200C). After the LDPE was in a molten state and the torque was stable, put in three-layer shell nucleus microcapsules, the antioxidant, and DCP and mixed for 20 min. After that, the homogeneous composite material was removed and cut into small particles. The antioxidant and DCP accounted for 0.3 wt% and 1.8 wt% of the total mass of LDPE, and the amount of doped microcapsules accounted for 0.0 wt%, 1.0 wt%, 2.0 wt%, 3.0 wt%, 4.0 wt%, and 5.0 wt% of the total mass of LDPE, respectively. Five groups of microcapsules/XLPE composites doped with different contents of microcapsules were prepared.

Firstly, the mixed composites were melted and molded in a plate vulcanization machine at 110 °C. After heating for 15 min, the pressure was increased by 5 MPa every 5 min until 15 MPa. The above-mentioned molten material was subjected to a 35 min cross-linking reaction in a 175 °C, 15 MPa plate vulcanizer, after which the thermo-compression molded materials were fully cooled and dried to obtain three-layer shell nucleus microcapsules/XLPE composites.

### 2.3. Structural Characteristics of the Three-Layer Shell Nucleus Microcapsules

The microstructure of the three-layer shell nucleus microcapsules was observed using an optical microscope (OM) and a scanning electron microscope (SEM). The equipment used for an SEM was SU8020 from Hitachi, Chiyoda-ku, Tokyo, Japan, and the equipment used for an OM was XSP-8CE from Changfang, Shanghai, China. Additionally, in order to prove the preparation effect, a Fourier spectrometer was used to analyze the chemical composition of the three-layer shell core microcapsules and determine the functional groups present in the sample. The equipment used was an FTIR-6100 Fourier Transform Infrared Spectrometer from JASCO, Hachioji, Tokyo, Japan.

In addition, to ensure the infrared absorption peak intensity of the powdered microcapsules, the microcapsules were mixed with potassium bromide for grinding treatment; the mass ratio was 1:200 and tableted in a 25 MPa tablet press. The test wavenumber range is 400–4000 cm^−1^, the resolution is 4 cm^−1^, the number of scans is 30 times, and the transmission mode was selected as the test mode.

### 2.4. Typical Characteristics of the Three-Layer Shell Nucleus Microcapsules/XLPE Composites

The effects of different concentrations of three-layer shell nucleus microcapsules on the typical properties of composites were investigated by crystallinity, AC breakdown characteristics, dielectric constant, and space charge. In addition, the influence mechanism of three-layer shell nucleus microcapsules on the properties of the XLPE matrix was discussed, and the optimum proportion of microcapsule doping was obtained.

In this paper, the differential scanning calorimeter (DSC822e) produced by Mettler Toledo Instruments Co., Ltd. in Zurich, Switzerland was used to test the XLPE composites with different contents of microcapsules and calculate the crystallinity. The internal structure of XLPE material can be divided into crystalline and amorphous regions. The crystallinity is usually used to represent the polymer’s crystalline region ratio. The present aging theory shows that the growth and development of water trees are mainly concentrated in the amorphous region of the material, so the crystallinity of composites has a great influence on the development trend of the water tree branch. The temperature range used in this experiment was −25 °C~150 °C, the nitrogen flow rate was 150 mL/min, and the mass of the specimen used was 5~10 mg.

The breakdown performance of XLPE is one of the influential indicators that characterize its breakdown strength. So, this paper uses an AC breakdown test platform to test the breakdown field strength of composite insulation materials at microcapsule doping concentrations of 0.0 wt%, 1.0 wt%, 2.0 wt%, 3.0 wt%, 4.0 wt%, and 5.0 wt%. The influence mechanism of the microcapsule system on the breakdown field strength of XLPE was also explored. The entire measurement device needs to be filled with dimethyl silicone oil to prevent surface discharge interference. During the experiment, the test was carried out by applying pressure step by step. The pressurization was swiftly stopped when the breakdown occurred, and the overcurrent reset was performed. The corresponding voltage was recorded. The above operation was repeated until 15 voltage values were recorded for each group XLPE composite doped with different contents of microcapsules.

This paper uses broadband dielectric spectrum to analyze the dielectric properties of composite insulation materials at microcapsule doping concentrations of 0.0 wt%, 1.0 wt%, 2.0 wt%, 3.0 wt%, 4.0 wt%, and 5.0 wt%. The equipment used was an Alpha-A broadband dielectric spectrum analyzer from Novocontrol, Frankfurt, Hesse, Germany. Before testing, the sample needs to be cleaned with absolute ethanol to remove the influence of impurities; it must be dried to avoid low-frequency dispersion caused by moisture. The temperature during the test was set to 30 °C, and the frequency was 10^−1^~10^6^ Hz.

The space charge acquisition system designed by pulse electroacoustic (PEA) was used for space charge testing of microcapsules/XLPE composites. Before the test, the thickness of the specimen was measured, and the required applied voltage was calculated. During the test, the temperature was set to 30.5 °C, the voltage applied was 40 kV/mm, and the pressurized polarization process lasted 2400 s.

### 2.5. Water Tree Aging Test

**Figure 2:**
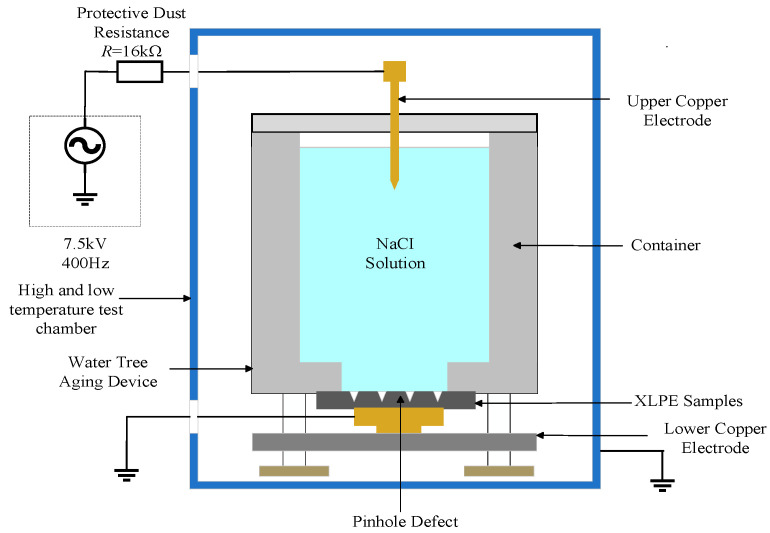
Simple structure diagram of the aging experimental platform.

A set of square microcapsules/XLPE composites was prepared using the flatbed vulcanizing machine, and pinholes were made in the central region to simulate water tree aging defects. The cut cylindrical polypropylene tube was fixed to the specimen, to which the configured sodium chloride solution was added, and a copper electrode was inserted. After connecting the circuit, the power supply was turned on for 30 days of the aging experiment. Figure 2 presents the simple structure diagram of the aging experimental platform.

### 2.6. Polarization/Depolarization Current Test

The depolarization current of the microcapsules/XLPE composites before and after aging was tested using the PDC method. A thin layer of vaseline reagent needed to be applied to the XLPE specimen and overlaid with aluminum foil during testing. In addition, the PDC test circuit adopted a three-electrode system to decrease the influence of the leak current. The experimental circuits used single-point grounding to prevent weak currents from returning to the ground line and affecting test accuracy.

## 3. Results and Discussion

### 3.1. Characterization of the Three-Layer Shell Nucleus Microcapsules

#### 3.1.1. Microscopic Observation of Three-Layer Shell Nucleus Microcapsules

**Figure 3:**
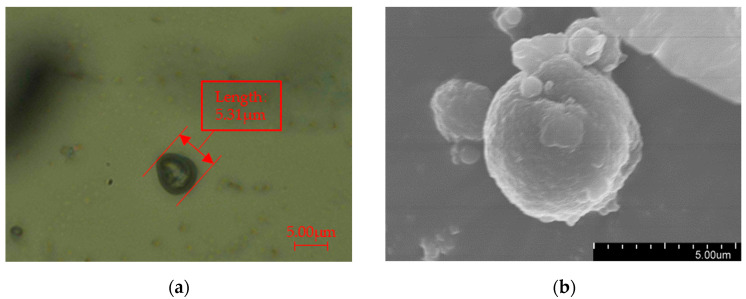
Microscopic morphology of a three-layer shell nucleus microcapsule: (**a**) OM diagram of a three-layer shell nucleus microcapsule; (**b**) SEM diagram of a three-layer shell nucleus microcapsule.

**Figure 4:**
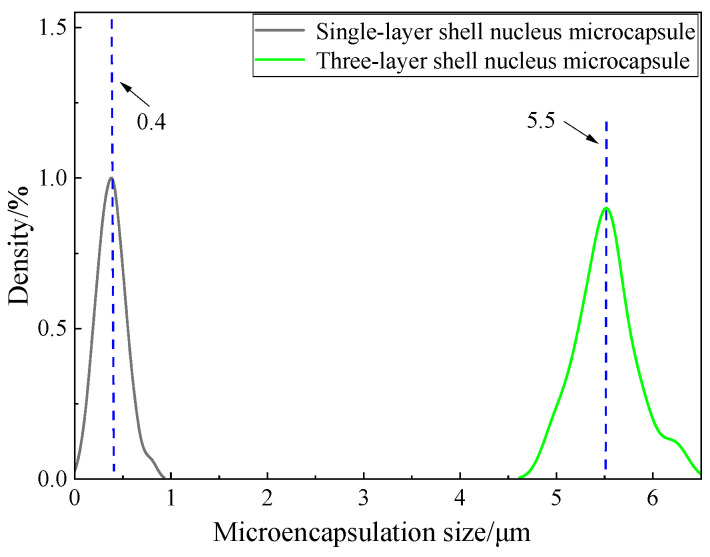
Particle size distribution of microcapsules.

The morphology of the three-layer shell nucleus microcapsules was observed by an OM and an SEM. The pictures obtained are shown in Figure 3. The microcapsules are spherical as a whole, with a complete shape and no damage. There are a series of depressions on the surface. The rough shell layer can enhance the compatibility between the microcapsules and the XLPE matrix, forming a composite material with a stronger bonding force and better performance. The microcapsule shell is complete, which shows that the shell layer has good mechanical properties and has a good supporting and protective effect on the microcapsule core material. The particle size distribution was obtained by statistically organizing the microcosmic morphology of the microcapsules. The statistical distribution is shown in Figure 4. The particle size of single-layer shell nucleus microcapsules was stable at approximately 0.4 μm, while three-layer shell nucleus microcapsules were stable at approximately 5.5 μm. The particle size of three-layer shell nucleus microcapsules was significantly larger than that of the single-layer shell nucleus microcapsules, which proved that the prepared microcapsules have a three-layer core-shell structure.

#### 3.1.2. Chemical Properties of the Three-Layer Shell Nucleus Microcapsules

**Figure 5:**
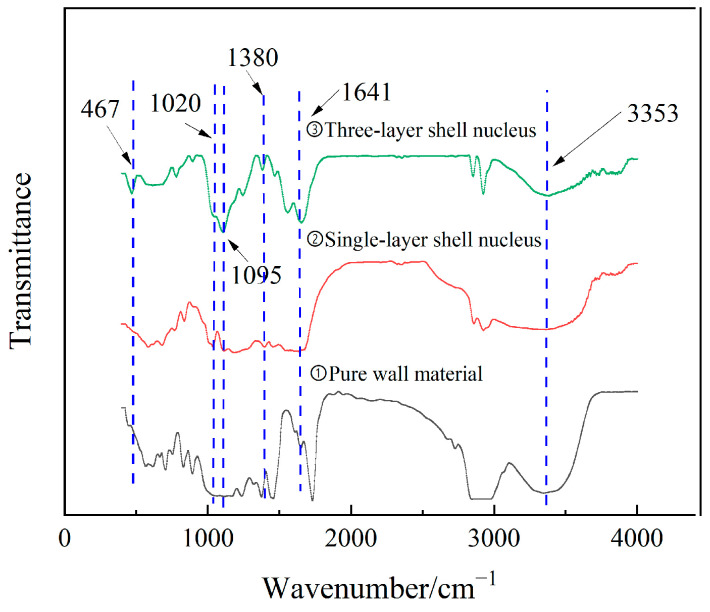
Infrared Spectra of microcapsules with different structures.

Figure 5 illustrates the infrared spectrum analysis results of microcapsules with different shell nucleus structures. As can be seen from the figure, the absorption peaks at different positions represent different chemical bonds. The pure wall material and microcapsules have obvious infrared characteristic absorption peaks at 3353 cm^−1^, 1641 cm^−1^, and 1020 cm^−1^. Among them, 3353 cm^−1^ is the stretching vibration absorption of the N-H and O-H overlapping peak. The broad and strong absorption peak here is the result of the hydrogen bonding effect in the UF molecular structure; 1641 cm^−1^ is the stretching vibration absorption peak of C=O in the UF amide bond, and the C-O stretching vibration absorption peak at 1020 cm^−1^ is due to the UF in Caused by primary alcohols produced during hydroxymethylation. These three infrared characteristic peaks prove that the microcapsules have UF wall materials.

Compared with pure wall material, both three-layer shell nucleus microcapsules and single-layer shell nucleus microcapsules exhibit characteristic infrared absorption peaks at 1380 cm^−1^, which is the vibration absorption peak of S=O, indicating the presence of the catalyst LABSA inside the microcapsules. Compared with pure wall material and single-layer shell nucleus microcapsules, the three-layer shell nucleus microcapsules show characteristic infrared absorption peaks at 467 cm^−1^ and 1095 cm^−1^, where the peak at 467 cm^−1^ is the stretching vibration absorption peak of Si-O, and the peak at 1095 cm^−1^ is the anti-symmetric stretching vibration peak of Si-O-Si, indicating that the three-layer shell nucleus microcapsules contain repair fluid DTMS and are coated with SiO_2_ on the surface.

### 3.2. Typical Performance of the Microcapsules/XLPE Composites

#### 3.2.1. Crystallization Characteristics

**Figure 6:**
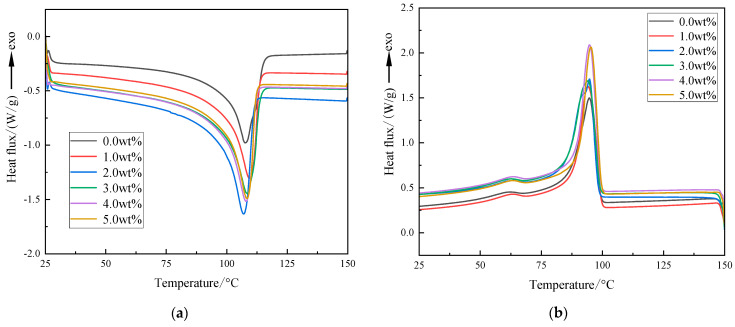
DSC test result of the three-layer shell nucleus microcapsules/XLPE composites: (**a**) The melting process; (**b**) The crystallizing process.

The DSC test results of the three-layer shell nucleus microcapsules/XLPE composites with different doping concentrations are shown in Figure 6, and the obtained crystallization and melting characteristic parameters are shown in Table 1. Among them, ω is the doping amount of microcapsules in composites, the melting peak Tm refers to the highest temperature peak observed during the melting process, and the crystallization peak Tc refers to the temperature corresponding to the fastest crystallization rate during the process of crystallization. The calculation formula of crystallinity is presented in Equation (1).

(1) $$X_{c}=\frac{\Delta H_{m}}{\Delta H_{100}}$$

In the formula: *X*_c_ represents the crystallinity of the composite material; Δ*H*_m_ represents the melting heat of the composite materials, Δ*H*_100_ represents the melting heat of ideal material when it is completely crystallized, where Δ*H*_100_ is 288 J/g for the XLPE material.

Figure 6 presents that the grain size distribution of the composites is narrow and then wide when the microcapsule content increases and the crystallinity increases and then decreases. The conclusion is that the doped microcapsule system will introduce a large amount of interface area, which plays a role in inducing nucleation during the recrystallization process of XLPE, reducing the nucleation-free energy of the matrix crystallization process, increasing the crystallization rate, and reducing the growth of spherulites rate, a large number of relatively uniform small spherulites are generated, resulting in an increase in the crystallinity of the material and a more uniform crystal size. However, when the concentration of microcapsules is too high, the interface area increases and the steric hindrance is enhanced; that is, the binding effect of the interface on the XLPE molecular groups is enhanced, which increases the viscosity of the system during the melting process, slows down the diffusion of XLPE molecular chains, and makes the material. The crystallization rate decreases, thereby reducing the crystallinity of the material. In addition, a larger doping concentration will reduce the distance between microcapsules, causing the unit cells formed at the interface of the microcapsules to be closer, which will hinder the growth of a single unit cell and cause multiple unit cells to contact each other to form large-sized cells crystallization, thereby reducing the crystallinity of the material and broadening the grain size distribution, resulting in thicker lamellae, broadening the grain size distribution and reducing the crystallinity of the material.

On the one hand, the nano-SiO_2_ can make the microcapsules and the XLPE matrix better integrate with each other and avoid microcapsule agglomeration. On the other hand, the nano-SiO_2_ in the polymer matrix can also be used as a heterogeneous nucleating agent, which promotes the orderly arrangement of XLPE molecular chains, accelerates the crystallization rate, and improves the crystallinity.

#### 3.2.2. AC Breakdown Characteristic

**Figure 7:**
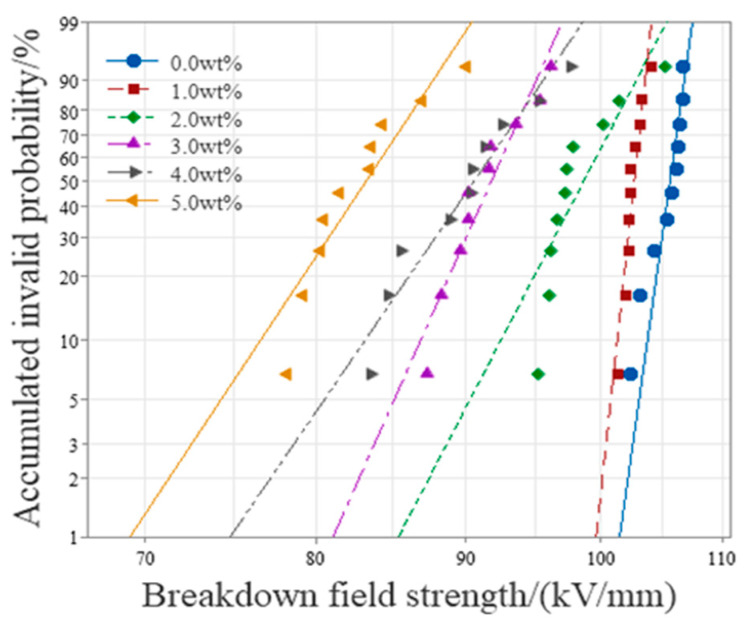
Weibull distribution of AC breakdown field strength of three-layer shell nucleus microcapsules/XLPE composite material.

In this paper, the breakdown field strength data of the obtained XLPE composites were processed using the Weibull statistical distribution. The statistical distribution map obtained by the analysis is shown in Figure 7. It is obvious that doping three-layer shell nucleus microcapsules into the XLPE matrix will reduce the insulation performance of composites. The breakdown field strength of microcapsules/XLPE composites was about 96.84% of the XLPE material without microcapsules when microcapsules were doped at 1.0 wt%. Compared to the XLPE material without microcapsules, the breakdown field strength change was minor, and the distribution was more concentrated. However, when the doping amount of microcapsules was 5.0 wt%, the breakdown field strength decreased to about 70% of the XLPE material without microcapsules, the breakdown field strength reduced significantly, and the distribution was relatively dispersed, which seriously affected the insulating property of XLPE material.

On the one hand, doped microcapsules will introduce a large number of polar group impurities, which are prone to ionization under high field strengths, increasing the concentration of carriers and aggravating the impact ionization process, thereby reducing the breakdown field strength of the composite material. On the other hand, the breakdown field strength of both the wall material and core material of microcapsules is lower than that of XLPE, so the overall breakdown field strength of the material after doping microcapsules will tend to decrease. The interface area between the microcapsule and the matrix may introduce physical defects, thereby forming a relatively concentrated low-density area. The defect structure will distort the electric field and form a relatively concentrated electric field in the low-density area, thereby generating new partial discharge points, exacerbating the material deterioration, and reducing the breakdown field strength. The interface area contains a large number of charge traps, which capture space charges. Although the mean free path of carriers can be shortened, energy is transferred to form hot electrons when electrons are captured by the traps, which can destroy macromolecular chains into small molecular structures. And a low-density area is formed near the electrode, where impact ionization is more likely to occur, leading to discharge breakdown. Too many microcapsules will cause their spacing to shrink or contact and the interface areas to overlap, forming new conductive channels, which will instead reduce the migration obstacles of carriers; that is, carriers can obtain more energy and bombard the molecular structure of XLPE leads to the destruction of the material structure, which is manifested as a reduction in the breakdown field strength. At the same time, the nano-SiO_2_ can better integrate three-layer shell nucleus microcapsules with the matrix, prevent the microcapsules from accumulating in the matrix, and reduce the large-scale defects formed by particle accumulation in the composite material.

#### 3.2.3. Dielectric Properties

**Figure 8:**
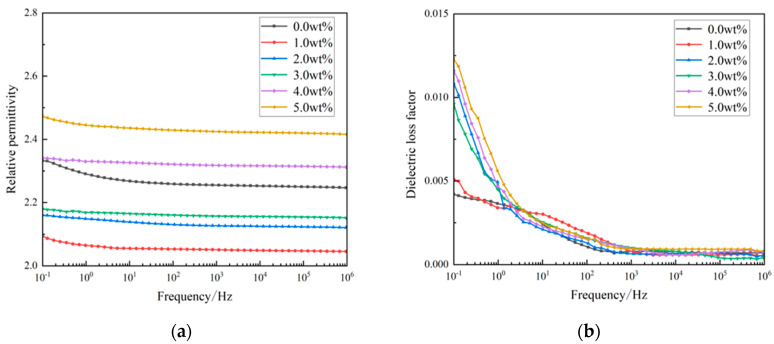
(**a**) Relative dielectric constants of the three-layer shell nucleus microcapsules/XLPE composites; (**b**) Dielectric loss factor of the three-layer shell nucleus microcapsules/XLPE composites.

The XLPE insulating material should have a small dielectric constant to decrease power dissipation during transmission. The dielectric constant versus frequency curves of the three-layer shell nucleus microcapsules/XLPE composites are shown in Figure 8. The relative permittivity and loss factor of all specimens reduce with the increase in frequency, mainly because both the electronic polarization and impurity polarization inside the material are able to synchronize with the external electric field at low frequencies. The dielectric loss of microcapsules/XLPE composites at low frequencies is mainly the conductivity loss, which leads to the higher loss factor in the low-frequency part.

With the increase in microcapsule doping amounts, the dielectric constant tends to decrease first and then increase. When the microcapsule doping amount is below 3.0 wt%, the relative permittivity of microcapsules/XLPE composites is smaller than XLPE material without microcapsules, and the loss factor of composites is higher than XLPE material without microcapsules. Microcapsules improve the crystallinity of XLPE material, and the dielectric constant of materials reduces with increasing crystallinity; this is because as the crystallinity of materials enhances, the free volume within the material decreases, the compactness of the material structure increases, the steering polarization decreases and therefore the dielectric constant of composites decreases. However, the addition of microcapsules also introduces many polar impurities within the matrix material, creating space charge polarization at the interface. Therefore, the dielectric constant of composites will increase when the doping content of microcapsules increases. In addition, at low doping concentration (especially 1 wt% concentration), compared with pure XLPE samples, the relative permittivity of composites decreases, and the loss factor does not change obviously, which could satisfy the fundamental requirements of XLPE insulated material operation.

#### 3.2.4. Space Charge Characteristics

**Figure 9:**
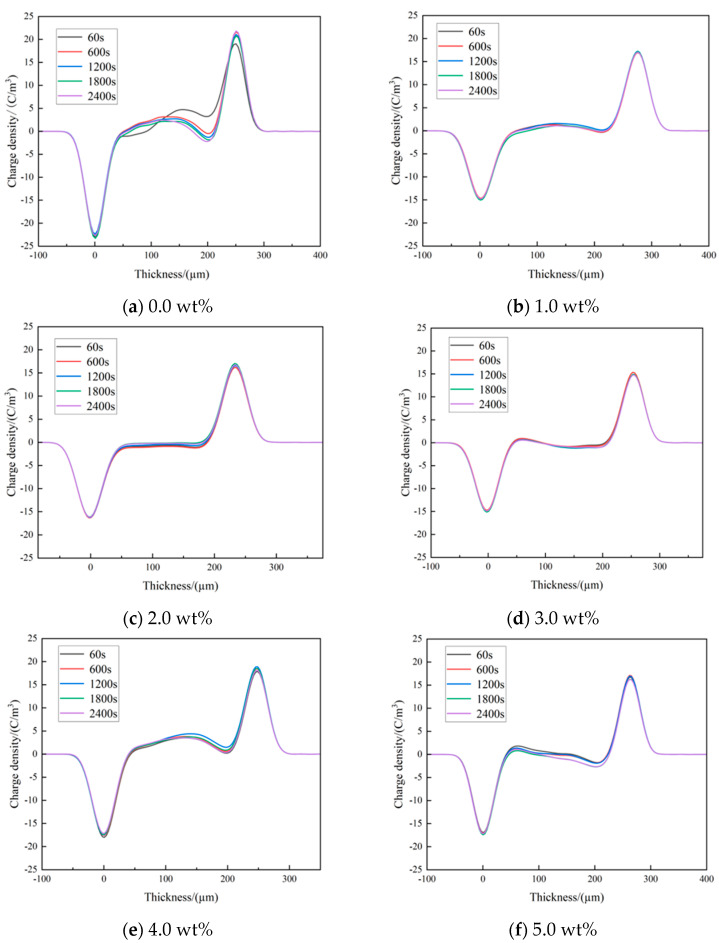
The space charge distribution map of the three-layer shell nucleus microcapsules/XLPE composite.

The electrons injected from the electrode and the impurity ions ionized within materials will accumulate in composites to form space charges. In this paper, the temperature was set to 30.5 °C during the experiment, the experimental field strength was 40 kV/mm, and the pressurized polarization time lasted for 2400 s. Figure 9 illustrates the space charge distribution of three-layer shell nucleus microcapsules/XLPE composites with different contents.

It can be seen from Figure 9a that the heteropolar space charge appears near the cathode and anode of the pure XLPE sample during the pressurization process, and its density increases with the pressurization time. This is because the impurity ions within the pure XLPE specimen dissociate at high field strengths, and the dissociated anions and cations move to the opposite electrode, resulting in the accumulation of heteropolar space charges around the electrodes. Meanwhile, in the presence of external voltage, the electrode injected many migrating carriers into the material to form a homopolar charge. The maximum space charge density accumulated at the cathode of the pure XLPE sample was 23.29 C/m^3^, and at the anode was 21.83 C/m^3^. After adding the microcapsule system, the heteropolar space charge accumulated near the cathode and anode of the three-layer shell nucleus microcapsules/XLPE composite was remarkably smaller than XLPE material without microcapsules during the pressurization process. When the doping concentration was 1% and 2%, there was almost an absence of space charge aggregation in composites, the homopolar charges injected by the electrode were also significantly reduced, and the space charge accumulation was improved, which indicated that the addition of the microcapsule system had an inhibitory effect on the space charge.

The incorporation of three-layer shell nucleus microcapsules alters the distribution of traps within the polymer matrix, with each microcapsule equivalent to a deep trap, and the introduced deep traps limit the space charge movement. In addition, the presence of microcapsules promotes carrier complexation, which helps to reduce space charge accumulation at the electrodes.

In summary, compared with pure XLPE materials, the crystallinity of the three-layer shell nucleus microcapsules/XLPE composites has increased, the dielectric constant has decreased, and the ability to suppress space charge has been significantly enhanced, but the insulation strength has reduced. By analyzing the typical characteristics of the three-layer shell nucleus microcapsules/XLPE composites, it is concluded that a concentration of 1.0 wt% is the optimum microencapsulation doping content for the composites while maintaining their properties.

### 3.3. Self-Healing Properties of Microcapsules/XLPE Composites for Water Tree Aging

#### 3.3.1. Microscopic Observations of Water Tree Ageing in Composites

**Figure 10:**
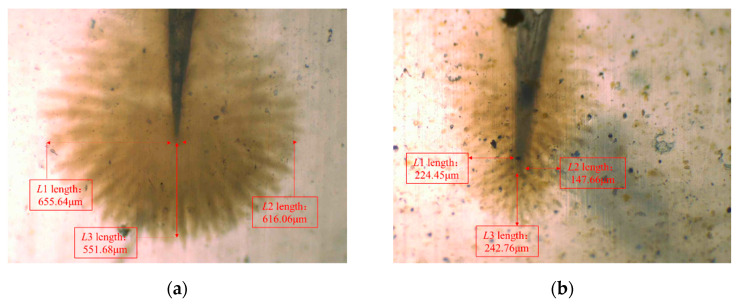
(**a**) The OM diagram of XLPE material without microcapsules after 30 days of aging. (**b**) The OM diagram of three-layer shell nucleus microcapsules/XLPE composites after 30 days of aging.

To clearly and intuitively observe the aging of three-layer shell nucleus microcapsules/XLPE composites, the aged samples were sliced, dyed, and then observed by an OM. The water tree micro-morphology of three-layer shell nucleus microcapsules/XLPE composites after aging for 30 days is shown in Figure 10.

According to Figure 10, it is evident that the water tree tends to develop toward the existence of microcapsules. It was observed that the water tree growth length of three-layer shell nucleus microcapsules/XLPE composites was significantly smaller than the XLPE material without microcapsules. The slightly larger dielectric constant of the microcapsules itself will affect the local electric field distribution within the material, thereby changing the expansion process of the water tree. The macroscopic manifestation is the development of attracted water tree. Water trees will develop on the microcapsules and break the capsule wall, thus consuming a certain amount of energy and inhibiting the development of water trees. The impurities and interfaces introduced by microcapsules will destroy the continuity of the original structure of the matrix, thereby hindering the expansion of water trees and limiting their size. Therefore, the overall size of the water tree within the composite decreases and tends to develop toward the microcapsule region. In addition, the water tree attracted by the microcapsules will cause the capsule wall to rupture, thereby triggering the self-healing behavior of the material. The repairing agents and catalysts in the microcapsules flow into the water tree channels under the action of capillary and infiltration, contact with water, and react to fill the damaged defects. At the same time, the nano-SiO_2_ enhances the mechanically interlocked ability of the microcapsules with the polymer substrate so that the two can better integrate, which avoids the agglomeration of the microcapsules in the matrix and further improves the repair rate.

**Figure 11:**
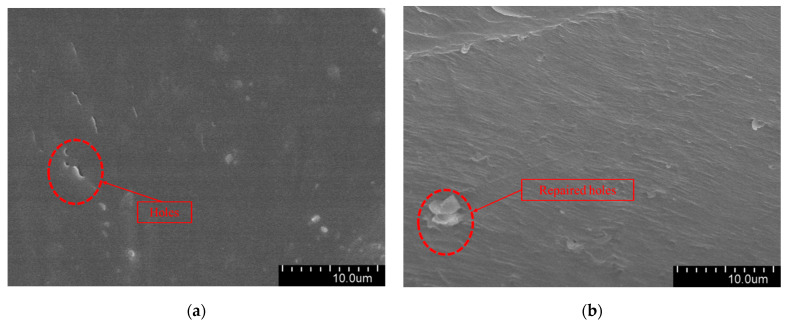
(**a**) The SEM diagram of XLPE material without microcapsules after 30 days of aging; (**b**) The SEM diagram of three-layer shell nucleus microcapsules/XLPE composites after 30 days of aging.

The aged three-layer shell nucleus microcapsules/XLPE specimens were quenched and observed by an SEM. Figure 11 shows the SEM image of the water tree region. This is shown in Figure 11a, where there are apparent pores inside the XLPE sample without microcapsules after water tree aging, which proves that the water tree is generated in the material during the aging experiment. Moreover, Figure 11b shows that some pores in microcapsules/XLPE composites are filled, which proves that during the water tree aging process, the high voltage can break the capsule wall of the three-layer shell nucleus microcapsules and release the restoration liquid. The repair solution reacts with the water in the water tree under the catalytic effect to form organic matter to cover the pores.

Adding a microcapsule system is equivalent to introducing impurities into XLPE. On the one hand, many interface areas will be generated between the microcapsules and the polymer matrix, resulting in structural defects. On the other hand, the dielectric characteristics of microcapsules and XLPE are different. This makes the area where the microcapsule exists more prone to water tree aging. When the growing water tree branches encounter and break microcapsules, the development of water tree branches is inhibited, and the core material in the microcapsules flows into the aging area and fully reacts with the water in it. After the reaction, a new organic matter is formed, and the fundamental reaction equations are presented in (2), (3) and (4).

(2) $${\mathrm{PhMeSi}(\mathrm{OMe})}_{2}{+H}_{2}O\to \mathrm{PhMeSi}(\mathrm{OH})(\mathrm{OMe})+\mathrm{MeOH}$$

(3) $${\mathrm{PhMeSi}(\mathrm{OH})(\mathrm{OMe})+H}_{2}O\to {\mathrm{PhMeSi}(\mathrm{OH})}_{2}+\mathrm{MeOH}$$

(4) $$2\mathrm{PhMeSi}(\mathrm{OH})(\mathrm{OMe})\overset{\mathrm{Catalyst}}{\to }\mathrm{PhMe}(\mathrm{OMe})\mathrm{Si-O-SiPhMe}(\mathrm{OMe})+H_{2}O$$

In the formula, Me and Ph present methyl and phenyl. The water in the water tree area is consumed, and the silicone resin produced by the reaction can fill the tiny pores caused by aging, thereby restoring the insulation performance of XLPE composite materials. In addition, the electrical characteristic of silicone resin is close to the XLPE material, which makes it more compatible with the matrix and more suitable for the application requirements of XLPE cable.

**Figure 12:**
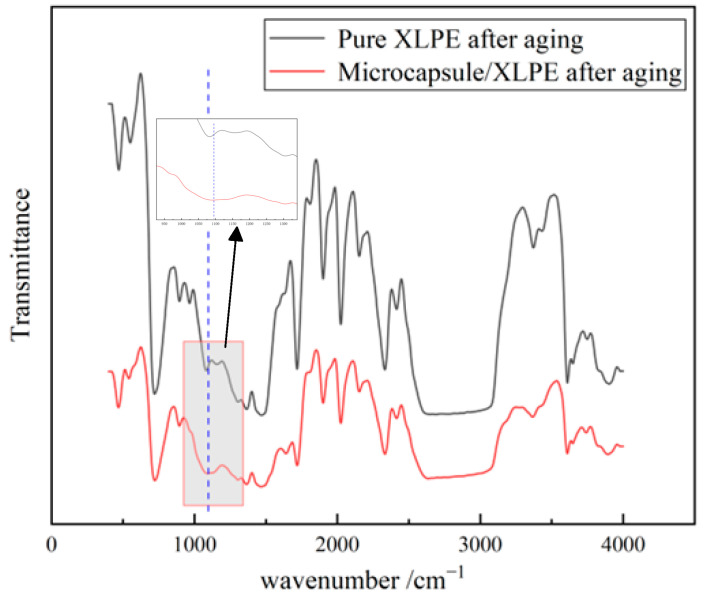
Infrared spectra of microcapsule/XLPE composite.

This paper performed Fourier transform infrared spectroscopy tests on aged pure XLPE specimens and aged microcapsule/XLPE specimens. The results are shown in Figure 12. According to the chemical composition characteristics of the organic silicone resin, the anti-symmetric stretching vibration peak of Si-O-Si at the wave number of 1095 cm^−1^ was selected to indicate that the organic silicone resin was formed in the water tree cavity.

In this paper, a microcapsule/cross-linked polyethylene composite is used to repair water tree damage. Compared with Zhou Kai’s injection of repair liquid into cross-linked polyethylene cables to repair water tree damage, Zhou Kai needs to pressurize and power off the cables, which seriously limits its scope of use [31]. The composite material in this paper can be repaired in the early stage of water tree generation, inhibit the development of water trees, restore the performance of the material, and prolong the service life of the insulating material.

#### 3.3.2. Dielectric PDC Response under Step Excitation

The dielectric time domain test is a method to reflect the dielectric parameters of the material by measuring the response of the step voltage excitation to the XLPE material. After applying DC voltage to XLPE samples, the relationship between polarization and depolarization current and time was tested, and the dielectric relaxation process in the time domain was studied.

The XLPE material is regarded as an infinite ideal plate electrode, and the electric field $\overset{\cdot }{E}(t)$ is applied to the isotropic dielectric between the parallel plates. The electric displacement vector is as follows:

(5) $$\overset{\cdot }{D}=\varepsilon_{0}\overset{\cdot }{E}+\overset{\cdot }{P}$$

According to the total current law, the full current density of the dielectric is:

(6) $$\overset{\cdot }{J}(t)=\sigma_{0}\overset{\cdot }{E}+\frac{\partial \overset{\cdot }{D}}{\partial t}=\sigma_{0}\overset{\cdot }{E}+\varepsilon_{0}\frac{\partial \overset{\cdot }{E}}{\partial t}+\frac{\partial \overset{\cdot }{P}}{\partial t}$$

In the formula: *σ*_0_ is the DC conductivity of the dielectric, S/m; *ε*_0_ is the vacuum dielectric constant (*ε*_0_ = 8.854 × 10^−12^ F/m); $\sigma_{0}\overset{\cdot }{E}$ is the conduction current; $\varepsilon_{0}\frac{\partial \overset{\cdot }{E}}{\partial t}$ is vacuum displacement current; $\frac{\partial \overset{\cdot }{P}}{\partial t}$ is the polarization current.

The polarization strength of the insulation can be expressed as follows:

(7) $$\overset{\cdot }{P}(t)=\varepsilon_{0}(\varepsilon_{\infty }-1)\overset{\cdot }{E}(t)+\varepsilon_{0}\int_{-\infty }^{t}f(t)\overset{\cdot }{E}(t-\tau )d\tau$$

In the formula, *ε*_∞_ is the relative dielectric constant of XLPE material at the optical frequency, *f*(*t*) is the dielectric response function of XLPE, and *τ* is the integral time constant.

Substitute (7) into (6). The full current density is as follows:

(8) $$\overset{\cdot }{J}(t)=\sigma_{0}\overset{\cdot }{E}+\varepsilon_{0}\varepsilon_{\infty }\frac{d\overset{\cdot }{E}(t)}{dt}+\varepsilon_{0}\frac{d}{dt}\int_{-\infty }^{t}f(t)\overset{\cdot }{E}(t-\tau )d\tau$$

When voltage *U*(*t*) is applied to XLPE material, the full current flowing through the material is:

(9) $$i(t)=C_{0}\frac{\sigma_{0}}{\varepsilon_{0}}U(t)+C_{0}\varepsilon_{\infty }\frac{dU(t)}{dt}+C_{0}\frac{d}{dt}\int_{-\infty }^{t}f(t)U(t-\tau )d\tau$$

In the formula, *C*_0_ is the vacuum geometric capacitance of the material, F.

The calculation method of *C*_0_ is as follows:

(10) $$C_{0}=\frac{\varepsilon_{r}S}{4\pi kd}$$

In the formula, *ε*_r_ is the relative dielectric constant of the material; *S* is the direct area between the two electrode plates, m^2^; *k* is the electrostatic force constant; and *d* is the distance between the plates, m.

If the XLPE material is completely discharged before pressurization, the DC power supply *U*_0_ is connected when *t =* 0, and the polarization current flowing through the material is:

(11) $$i_{\mathrm{pol}}(t)=C_{0}U_{0}\left[\frac{\sigma_{0}}{\varepsilon_{0}}+\varepsilon_{\infty }\delta (t)+f(t)\right]$$

In the formula, *i*_pol_(*t*) is the polarization current of the material, A, and *δ*(*t*) is the impact function.

Since the change in the impulse current amplitude during the polarization process is difficult to measure, it is generally not considered, that is:

(12) $$\varepsilon_{\infty }\delta (t)\approx 0$$

When *t = t*_a_ (*t*_a_ is the charging time), the XLPE material is grounded and discharged, and the depolarization current flowing through the material is:

(13) $$i_{\mathrm{depol}}(t)=C_{0}U_{0}[f(t)-f(t+t_{a})]$$

In the formula, *i*_depol_(*t*) is the depolarization current of the material, A, and *t*_a_ is the charging time, s.

*f*(*t*) is a monotonically decreasing function. When the charging time is very long, *f*(*t + t*_a_) in the above equation can be ignored. Therefore, we have the following:

(14) $$f(t)\approx \frac{i_{\mathrm{depol}}(t)}{C_{0}U_{0}}$$

If ta is long enough, the polarization current is approximately equal to the sum of the depolarization current and the conduction current. The DC conductivity expression of XLPE material is obtained by combining Equations (11), (12) and (14):

(15) $$\sigma_{0}=\frac{\varepsilon_{0}}{C_{0}U_{0}}[i_{\mathrm{pol}}(t)-i_{\mathrm{depol}}(t)]$$

#### 3.3.3. XLPE Material Equivalent Modeling and Aging Factors

**Figure 13:**
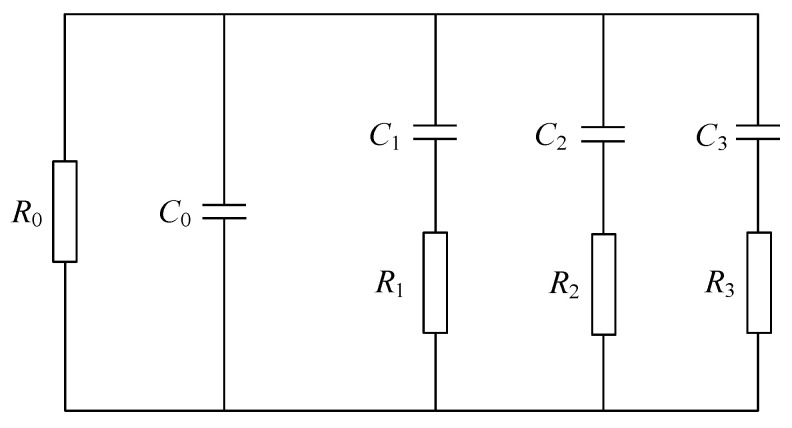
Three-branch extended Debye model of XLPE material.

XLPE material aging occurs, a large number of defects will appear within the material, each defect can be equated to an RC series branch, and water tree aging XLPE material can be equated with the three-branch Debye model shown in Figure 13. In the figure, *R*_0_ and *C*_0_ are the insulation resistance and geometric capacitance of XLPE material, respectively; *R*_1_ and *C*_1_ in the first branch are the parameters to characterize the polarization of XLPE material, which are related to the polarization phenomenon of its body; *R*_2_ and *C*_2_ in the second branch are the branch parameters that characterize the interface polarization between the amorphous region and the crystalline region; *R*_3_ and *C*_3_ in the third branch are related to the polarization of metal salts, polar groups and hydrated ions in XLPE materials.

The depolarization current obtained by the PDC test is decomposed into a third-order exponential decay function:

(16) $$i_{\mathrm{depol}}(t)=I_{0}+\sum_{i=1}^{3}a_{i}e^{-\frac{t}{\tau_{i}}}$$

In the formula, *a*_i_ can reflect the trap density in the medium; *τ*_i_ can reflect the trap depth in the medium; and *I*_0_ is the steady-state value of the depolarization current, A.

In the three-branch extended Debye model of XLPE material, the time constants of the first and second branches are small, and the time constants of the material are almost unchanged during normal operation. However, when the water tree aging occurs inside the material, the third branch time constant *τ*_3_, representing the polarization of metal salts, polar groups, and hydrated ions in the material, changes greatly. Due to the particularity of the water tree aging of the material, the aging factor *A*_f_ calculated by the ratio of *Q*(*τ*_2_) and *Q*(*τ*_3_) can be used to characterize the aging degree of the material. *Q*(*τ*_2_) represents the influence of the interface polarization between the amorphous region and the crystal region in the XLPE material. *Q*(*τ*_3_) represents the influence of the polarization of ions and water molecules. The calculation formula is as follows:

(17) $$A_{f}=\frac{Q(\tau_{3})}{Q(\tau_{2})}$$

In the formula, *A*_f_ is the aging factor of XLPE material. The larger the value is, the higher the aging degree of the material is. The expressions of *Q*(*τ*_2_) and Q(*τ*_3_) are as follows:

(18) $$Q(\tau_{2})=a_{1}\tau_{1}+a_{2}\tau_{2}(1-\frac{1}{e})+a_{3}\tau_{3}(1-e^{-\frac{\tau_{2}}{\tau_{3}}})$$

(19) $$Q(\tau_{3})=a_{1}\tau_{1}+a_{2}\tau_{2}(1-e^{-\frac{\tau_{3}}{\tau_{2}}})+a_{3}\tau_{3}(1-\frac{1}{e})$$

#### 3.3.4. PDC Analysis of the Microcapsules/XLPE Composites

**Figure 14:**
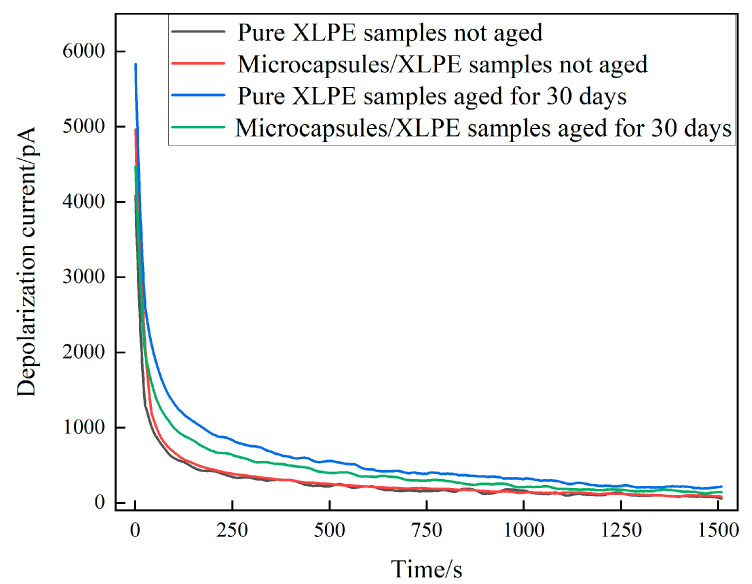
Depolarization current of the microcapsules/XLPE samples.

The PDC test was carried out on the unaged microcapsules/XLPE sample and the aging microcapsules/XLPE sample. Figure 14 presents the results of depolarizing currents of microcapsules/XLPE composites, from which we can see that the initial and steady value of depolarizing currents of the XLPE material without microcapsules and three-layer shell nucleus microcapsules/XLPE specimens increased obviously after the aging experiment, which indicates that the aging defects lead to the degradation of the insulating strength of the XLPE specimens. This is because internal traps of XLPE samples increase after water tree aging, and the polarization characteristics are enhanced. In addition, comparing the two groups of unaged samples, it can be seen that the depolarization current of the microcapsules/XLPE composite is faintly larger than the XLPE material without microcapsules, which indicates that microcapsules will exacerbate the interfacial polarization of composites and cause depolarization currents to increase. Nevertheless, at 1.0 wt% of microcapsule doping content, the depolarization current of composites changes little, which has no significant impact on the performances of the sample and could meet the requirements of regular operation.

In addition, compared with pure XLPE samples after the aging experiment, the initial value and steady value of depolarization current of microcapsules/XLPE composites are lower after the aging experiment, which demonstrates that microcapsules can fix aging defects of XLPE composite materials, fill the holes caused by water tree aging. Meanwhile, the electrical characteristics of the silicone resin generated by the reaction are similar to the matrix polymer, which reduces polarization currents within composites and restores their insulating properties. Its steady-state leakage current can be restored to about 75% of the initial state.

In order to analyze the change in the DC conductivity of XLPE material before and after water tree aging and self-healing, the stable values of polarization current and depolarization current are used to approximately calculate the conductivity of the material, and the results are substituted into Equation (15). The DC conductivity of unaged pure XLPE sample, unaged microcapsule/XLPE sample, aged pure XLPE sample, and aged microcapsule/XLPE sample can be obtained by calculation, as shown in Table 2.

From Table 2, it can be seen that the DC conductivity of the unaged pure XLPE sample and the unaged microcapsule/XLPE sample is close. With the increase in aging time, the conductivity of the two samples also increases, but the conductivity of the XLPE sample doped with microcapsules is lower than that of the pure XLPE sample. The analysis shows that after the water tree aging of the XLPE sample, a large number of structural defects appear in the material, and more space charges accumulate at the interface between the water tree and the material, causing the interface polarization. Therefore, the DC conductivity of the material also increases after the water tree aging of the material. The repair agent plays a filling role in the aging material, reduces the structural defects in the material, suppresses the electric field distortion effect in the water tree area, and thus reduces the DC conductivity of the aging material. At the same time, the dielectric properties of the generated organic polymer are close to those of the XLPE matrix, which uniforms the high field strength at the internal defects of the material, restores the insulation performance of the material, and exhibits good self-healing ability.

In order to more intuitively show the aging degree of the composite material and the self-healing performance of the microcapsule/XLPE material, the aging factor is used as the basis for judging the aging degree of the material. Since defects are inevitably caused inside the XLPE sample during the preparation process, the aging factor of the sample is higher than that of the new cable. The depolarization current is substituted into Equation (16) for the third-order attenuation exponential function fitting, and the results are substituted into Equation (17) to Equation (19) to obtain the aging factor of the XLPE sample. The result is approximately regarded as the water tree aging degree of the sample. The aging factors of the unaged pure XLPE sample, the unaged microcapsule/XLPE sample, the aged pure XLPE sample, and the aged microcapsule/XLPE sample are shown in Table 3.

It can be seen from Table 3 that the aging factors of the unaged pure XLPE sample and the unaged microcapsule/XLPE sample are close. With the increase in aging time, the aging factors of the two samples also increase. However, the aging factor of the XLPE sample doped with microcapsules is lower than that of the pure XLPE sample, indicating that the defect structure of the water tree is filled by the organic polymer generated by the microcapsules. Because its dielectric properties are similar to those of the XLPE matrix, the deep traps at the water tree defects are weakened, and the discharge energy accumulated at the defects is released, thereby reducing the interfacial polarization effect, insulation degradation process, and the aging factor of the material. The insulation performance of the material is restored.

## 4. Conclusions

When the doping amount of the three-layer shell nucleus microcapsules is 1.0 wt%, the breakdown field intensity of microcapsules/XLPE composites is close to the XLPE material without microcapsules, and other typical electrical properties are improved. Therefore, the 1.0 wt% concentration is the best doping ratio for the composite to achieve self-healing while maintaining its performance. The nano-SiO_2_ on the surface of the three-layer shell nucleus microcapsules can enhance the mechanically inter-locked ability of the microcapsules with the polymer substrate so that the two can better integrate, strengthen the densification of the material, and further improve the re-pair efficiency.

The water tree branches are prone to develop in the direction of the area where the microcapsule exists, and breaking the microcapsules requires a lot of energy, so the microcapsule system can inhibit the growth of water trees. After the water tree aging of microcapsules/XLPE composites, the external and internal capsule walls of three-layer shell nucleus microcapsules are broken sequentially, and the repair solution flows into the aging area to react with water, which consumes the water inside the aging area and fill micro-holes generated by aging. The produced silicone resin repairs the aging area and restores the insulation properties of the composites.

## Figures and Tables

**Table 1:** Crystallization melting characteristic parameters and crystallinities.

Microcapsule Content/wt%	*T_m_*/°C	*T_c_*/°C	*X*_c_/%
0.0 wt%	107.6	94.6	33.73
1.0 wt%	109.5	94.1	37.31
2.0 wt%	107.0	94.8	37.92
3.0 wt%	108.5	94.0	38.23
4.0 wt%	108.0	94.6	38.62
5.0 wt%	108.3	95.3	37.57

**Table 2:** DC conductivity of microcapsules/XLPE samples.

Sample	DC Conductivity/(10^−15^ S/m)
unaged pure XLPE sample	5.06
unaged microcapsule/XLPE sample	10.15
aged pure XLPE sample	214.34
aged microcapsule/XLPE sample	78.26

**Table 3:** Aging factor of microcapsules/XLPE samples.

Sample	Aging Factor *A*_f_
unaged pure XLPE sample	2.14
unaged microcapsule/XLPE sample	2.18
aged pure XLPE sample	2.95
aged microcapsule/XLPE sample	2.66

## Data Availability

Data are contained within the article.
